# Increasing awareness of degenerative cervical myelopathy: a preventative cause of non-traumatic spinal cord injury

**DOI:** 10.1038/s41393-021-00711-8

**Published:** 2021-10-09

**Authors:** Carl M. Zipser, Konstantinos Margetis, Karlo M. Pedro, Armin Curt, Michael Fehlings, Iwan Sadler, Lindsay Tetreault, Benjamin M. Davies, On behalf of the AO Spine RECODE DCM Steering Committee, Mark Kotter, Benjamin Davies, Brian Kwon, Carl Zipser, Shekar Kurpad, Vafa Rahimi-Movaghar, Bizhan Aarabi, James Harrop, James Guest, Sukhvinder Kalsi-Ryan, Angus McNair, Julio Furlan, Iwan Sadler, Delphine Houghton, Ellen Sarewitz, Julia Carter, Margot Miller, Timothy Boerger, Paige Howard, Shirley Widdop, Carla Salzman, Jamie Milligan, Geno J. Merli, Robert Chen, Jefferson R. Wilson, Ricardo Rodrigues-Pinto, Members of the Diagnostic Criteria Working Group, Ricardo Rodrigues-Pinto, Jamie R. F. Wilson, Nitin Kimmatkar, Jamie Milligan, Benjamin Davies, Ratko Yurac, Lucy Cameron, Carl Zipser, Mohamed Abdel-Wanis, Ellen Sarewitz, Bruno Lourenço Costa, Shirley Widdop, Michael Betz, Oke Obadaseraye, Karlo Pedro, Lianne Wood

**Affiliations:** 1 Spinal Cord Injury Centre, Balgrist University Hospital, Zurich, Switzerland; 2 Department of Neurosurgery, Icahn School of Medicine at Mount Sinai, New York, NY USA; 3 Division of Neurosurgery, Department of Neurosciences, University of the Philippines-Philippine General Hospital, Manila, Philippines; 4 Division of Neurosurgery, University of Toronto, Toronto, ON Canada; 5 Myelopathy Support, Myelopathy.org, Cambridge, UK; 6 Department of Neurology, NYU Langone Health, Graduate Medical Education, New York, NY USA; 7 Department of Neurosurgery, University of Cambridge, Cambridge, UK; 8 Myelopathy.org, International Charity for Degenerative Cervical Myelopathy, Bristol, Great Britain; 9 Vancouver Spine Surgery Institute, Department of Orthopedics, The University of British Columbia, Vancouver, BC Canada; 10 Department of Neurosurgery, Medical College of Wisconsin, Wauwatosa, WI USA; 11 Department of Neurosurgery, Sina Trauma and Surgery Research Center, Tehran University of Medical Sciences, Tehran, Iran; 12 Department of Neurosurgery, University of Maryland School of Medicine, Baltimore, MD USA; 13 Department of Neurological Surgery, Thomas Jefferson University, Philadelphia, PA USA; 14 Department of Neurosurgery and The Miami Project to Cure Paralysis, The Miller School of Medicine, University of Miami, Miami, FL USA; 15 KITE|Toronto Rehab|University Health Network. Clinic/Scientific Lead - Rocket Family Upper Extremity Clinic-Lyndhurst Site, Toronto, ON Canada; 16 Bristol Centre for Surgical Research, University of Bristol, Bristol, Great Britain; 17 KITE Research Institute, University Health Network, Toronto, ON Canada; 18 Department of Medicine, Division of Physical Medicine and Rehabilitation, University of Toronto, Toronto, ON Canada; 19 Department of Family Medicine, McMaster University, Hamilton, ON Canada; 20 Jefferson Vascular Center, Thomas Jefferson University, Philadelphia, PA USA; 21 Krembil Research Institute, Division of Neurology, Department of Medicine, University of Toronto, Toronto, ON Canada; 22 Spinal Unit (UVM), Department of Orthopaedics, Centro Hospitalar Universitário do Porto - Hospital de Santo António, Porto, Portugal; 23 Department of Neurosurgery, University of Nebraska Medical Center, Omaha, NE USA; 24 Government Medical College Nagpur, Nagpur, India; 25 Universidad del Desarrollo, Santiago, Chile; 26 University Hospitals NHS Foundation Trust, Addenbrooke’s or Rosie Hospital, Cambridge, Great Britain; 27 Sohag Faculty of Medicine, Sohag Governorate, Egypt; 28 Hospital de São Teotónio (Centro Hospitalar Tondela - Viseu, Epe), Coimbra, Portugal; 29 University Spine Center, Balgrist University Hospital, Zurich, Switzerland; 30 Orthopaedics and Trauma, Lagos, Nigeria; 31 Philippine General Hospital, University of the Philippines, Manila, Philippines; 32 Nottingham University Hospital, Nottingham, Great Britain

**Keywords:** Spinal cord diseases, Spinal cord diseases

## Abstract

Degenerative cervical myelopathy (DCM) is a common non-traumatic spinal cord disorder and characterized by progressive neurological impairment. Generally, it is still underdiagnosed and referral to spine specialists is often late, when patients already present with incomplete cervical spinal cord injury (SCI). To improve early diagnosis and accelerate referral, diagnostic criteria for DCM are required. Recently, AO Spine RECODE- DCM (REsearch Objectives and Common Data Elements for Degenerative Cervical Myelopathy) (aospine.org/recode), an international, interdisciplinary and interprofessional initiative, including patients with DCM, was funded with the aim to accelerate knowledge discovery that can change outcomes. In this perspective we advocate for the participation of SCI specialists in this process, where the expertise and perspective on this disorder and requirements for the diagnostic and therapeutic work up is well developed.

## Perspective

Degenerative cervical myelopathy (DCM) (historically termed “cervical spondylotic myelopathy” [CSM]) is the most common non-traumatic, progressive spinal cord disorder with an estimated 2% prevalence [1]. The disorder is indeed imprecisely and insufficiently characterized by neck and radicular pain, fine motor dysfunction, gait instability, and bladder dysfunction and for most lacks common diagnostic criteria [2]. If not recognized and treated timely, patients may eventually present as incomplete cervical spinal cord injury (SCI). This letter aims to raise awareness of these shortcomings in the neurological community and emphasize an ongoing initiative to improve clinical care and foster global research [3]. The neurological field should not be left out in this effort.

**Fig. 1:**
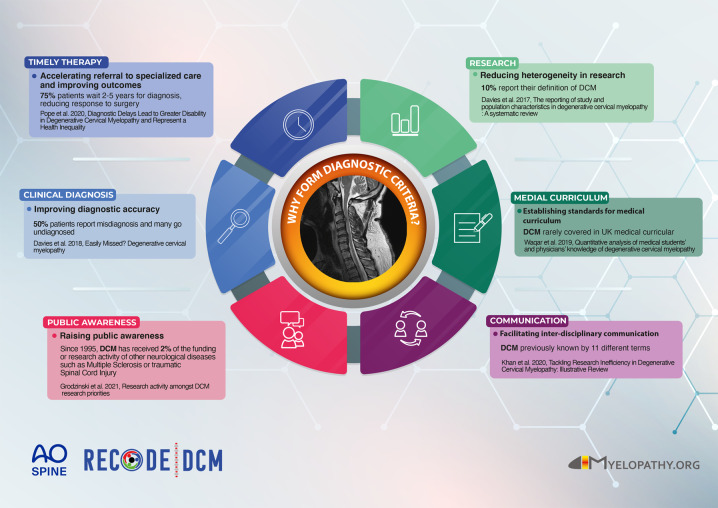
Infographic providing an overview why diagnostic criteria for DCM are needed. The panels summarize shortcomings in patient care and research which diagnostic criteria for DCM can help to overcome.

Due to a variety of symptoms, patients eventually get referred to different specialists, commonly orthopedics, neurosurgeons, neurologists, or physiotherapists. While surgical decompression of the encroached spinal cord is recommended in patients experiencing already moderate/severe, or progressive symptoms, the goal of enabling earlier and/or preventative treatment has now been defined as a priority research need. In its early stages, DCM is frequently underdiagnosed or misdiagnosed as carpal tunnel syndrome or peripheral neuropathy, until patients develop more severe impairments of upper and lower limb function urging the consideration of incomplete cervical SCI. Given that DCM is a progressive but preventable neurological condition, the delayed diagnosis and late referral for evaluation of surgical decompression, can lead to poorer neurological outcomes [4]. In addition, the pre-operative neurological status significantly influences post-operative recovery [5]. Therefore, an early diagnosis is important to achieve good clinical results. The diagnosis of DCM is currently based on clinical signs and symptoms, eventually complemented by cervical spine MRI. However, there is still no consensus on diagnostic criteria for DCM, leading to ambiguous, descriptive clinical diagnoses, and heterogenous definitions of DCM applied in clinical studies [6, 7]. Furthermore, as demonstrated in the UK, DCM is rarely covered in the medical curriculum [8]. AO Spine RECODE-DCM (REsearch Objectives and Common Data Elements for Degenerative Cervical Myelopathy) (aospine.org/recode) is an international, interdisciplinary, and interprofessional initiative, including patients with DCM, which aims to accelerate knowledge discovery that can change outcomes [9]. This has included the formation of research priorities such as the development of common diagnostic criteria. Alongside its importance for clinical care, a sensitive and specific set of diagnostic criteria is required to foster research, particularly for those studies aiming at investigating neuroprotective strategies. Diagnostic criteria for DCM can help to overcome several shortcomings in patient care and research (Fig. 1). The development of diagnostic criteria for DCM would benefit from the experiences of the neurological community; a UK cohort study identified 45% of cases are initially diagnosed by Neurologists, whilst Neurologists are familiar with their development and implementation [10]. Common criteria will help the dialog between neurologists, general practitioners (primary care providers), and spine specialists, and propagate the knowledge of red flags in DCM that require timely and specific actions, before established SCI. The development of diagnostic criteria involves both comprehensive diagnostic criteria for research and spine specialists, as well as easily applicable algorithms that speed up referral to cervical spine MRI and spine specialists. We have established an initial working group to act upon this opportunity (AO Spine RECODE DCM Diagnostic Criteria Incubator). If you are interested in contributing to this process, please contact us.
